# Supplementary material for: Three-dimensional characterization of developing and adult ocular vasculature in mice using in toto clearing
Source: Commun Biol. 2022 Oct 27;5:1135. doi: 10.1038/s42003-022-04104-2 (PMC9613908; doi:10.1038/s42003-022-04104-2)
Supplement: Supplementary file 2 — Supplementary Information-New [file 42003_2022_4104_MOESM2_ESM.pdf]

Supplementary Materials :

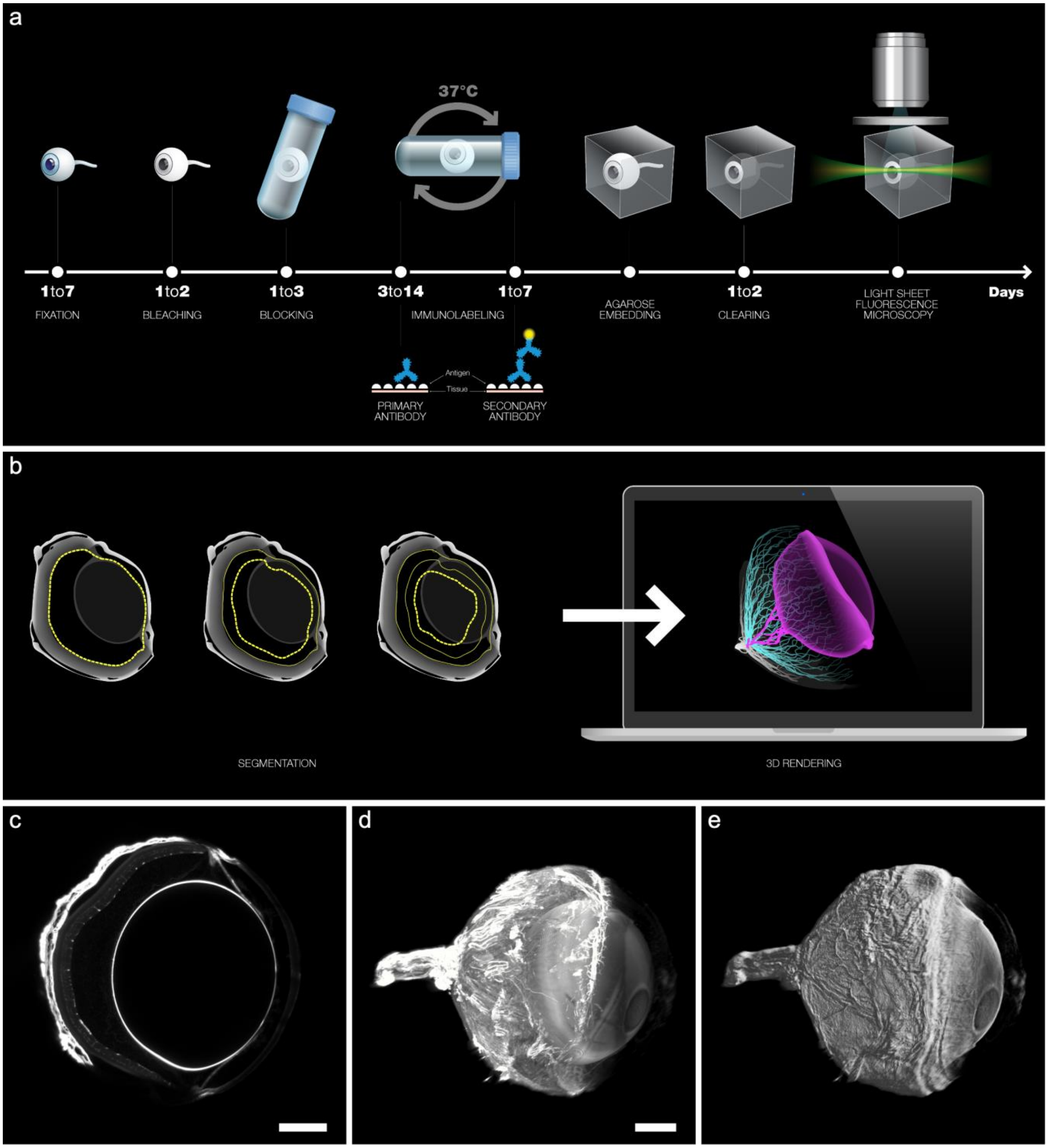

Supplementary Figure 1: clearing, 3D reconstruction and segmentation. a. clearing protocol and LSFM acquisition steps. After euthanasia, sample were fixed, bleached for depigmentation, processed through the immunohistochemistry protocol, then the clearing protocol (using iDISCO+ method), and finally imaged using LSFM. b. virtual segmentation

process. c to e: consecutive slices acquired by LSFM (c) are reconstructed in 3D and visualized in either opaque view (e) or raw signal (d). Scale Bars value: 500µm (e and d have the same scale bar).

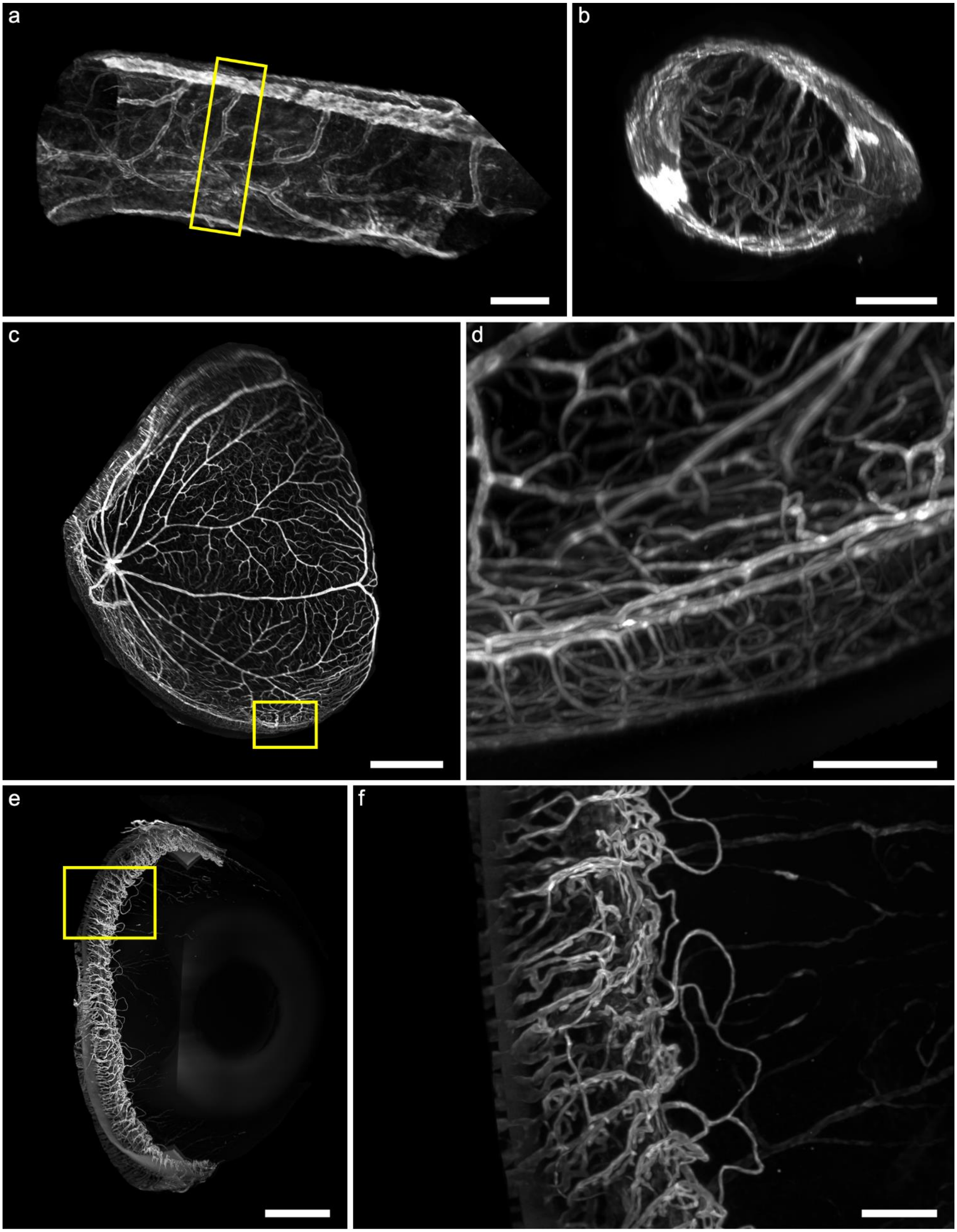

Supplementary Figure 2: **LSFM imaging of anti-colIV optic nerve (a and b) and retina (c and**

d) and anti-meca32 staining of the iris (e and f) (scale bars: a, b 100µm, c 500µm, d 100µm, e 500µm, f 100µm).

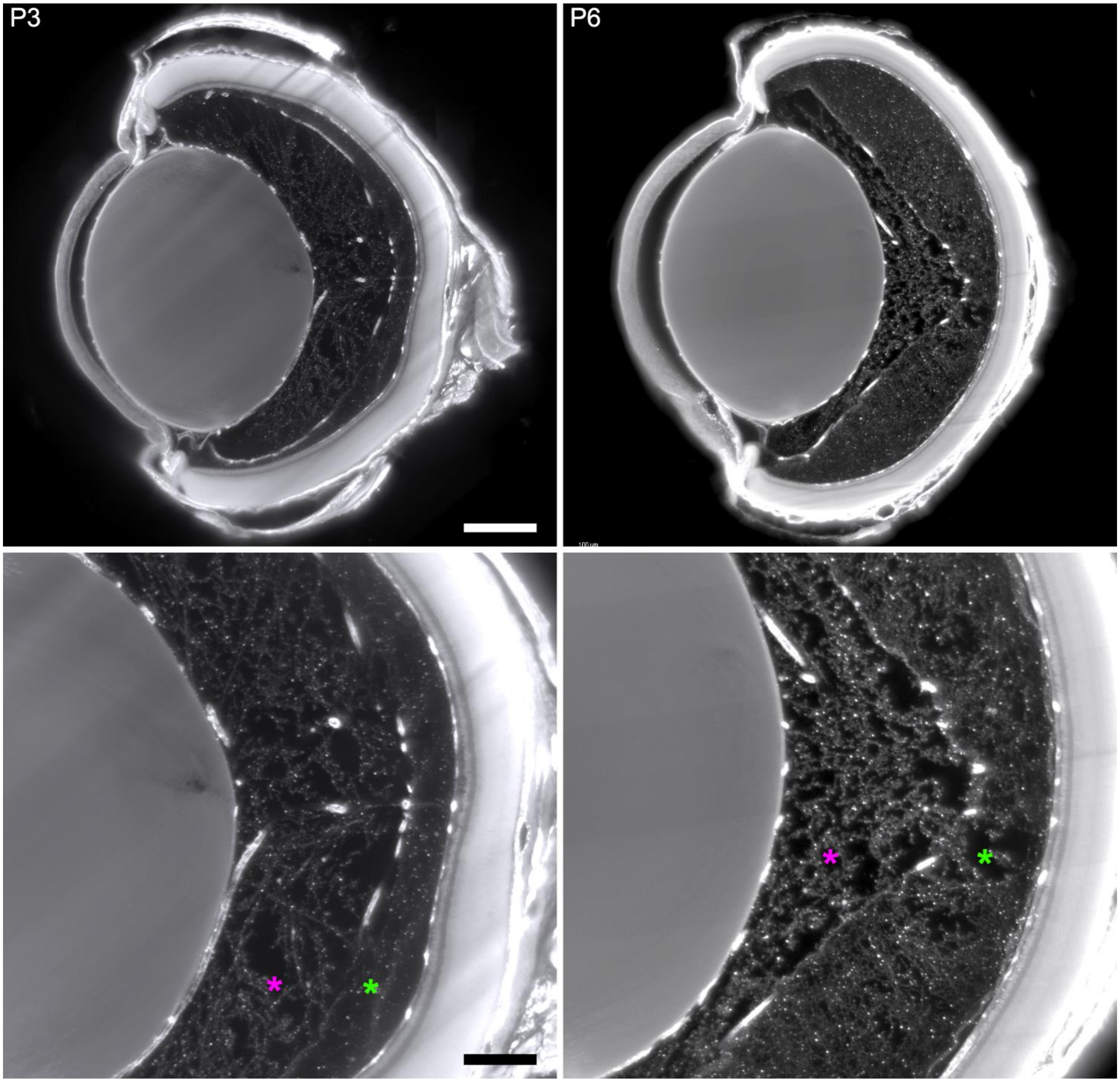

Supplementary Figure 3: Primary and secondary vitreous. Sagittal images of anti-CD31 stained entire eye at three (P3) and six (P6) postnatal days. The secondary vitreous grows between the two ages. The VHP is located in between the primary vitreous (magenta asterisk) and the secondary vitreous (green asterisk) (Scale bars first line: 200µm, second line 100 µm; VHP: vasa hyaloidea propria).

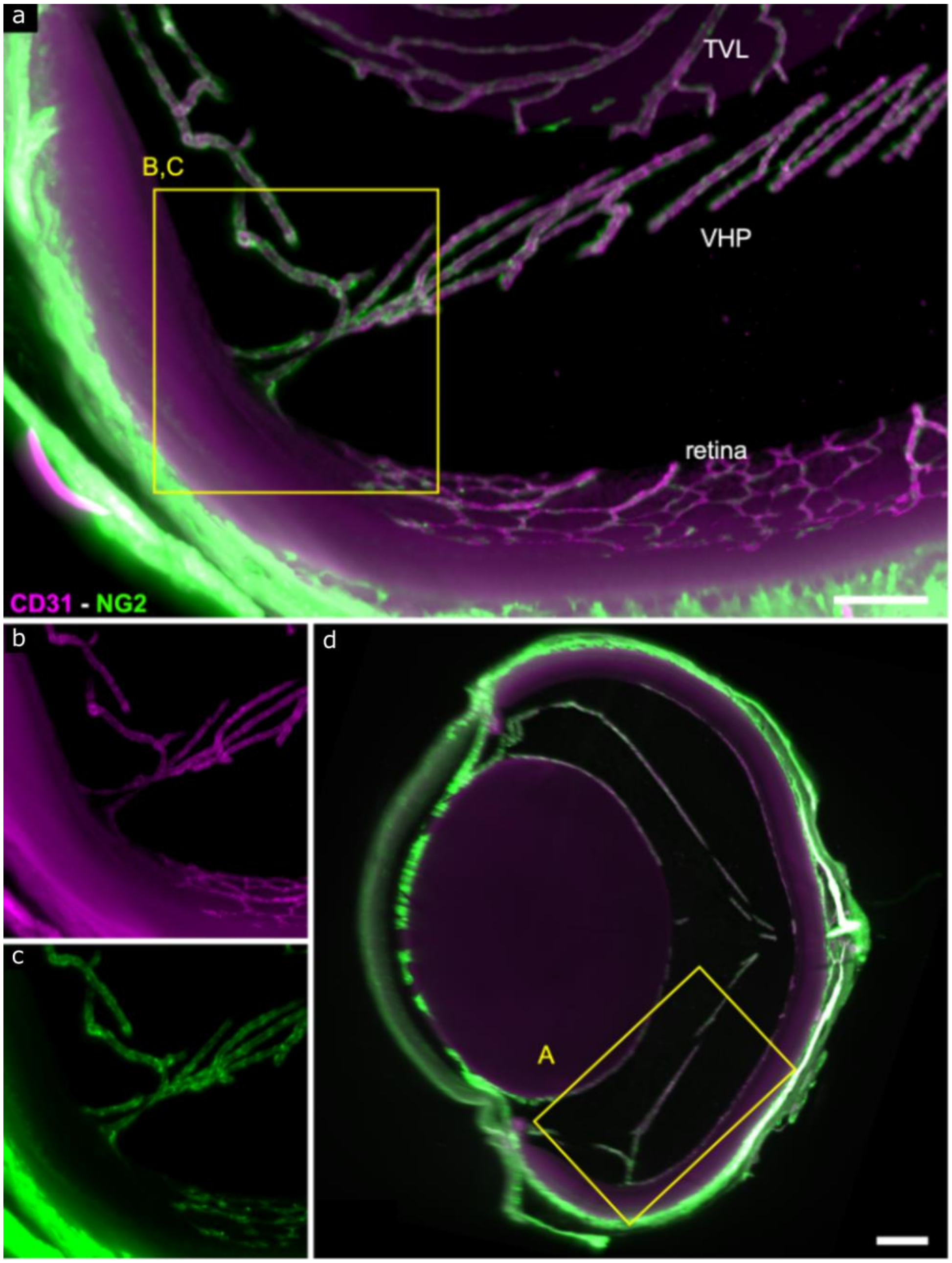

Supplementary Figure 4: Illustration of anatomical connections between de VHP and the retina at P6. At P6, connections between VHP and retina are present (a). These connections showed a weak CD31 staining (magenta, b), but a strong NG2 staining (green, c), similar to the phenotype of regressing vessels (see example in figure 4). Full view of a 65µm-depth optical slice of the P6 eye in d. All scale bars are 200µm, panels b and c correspond to the yellow square in a (same scale bar). abbreviations: P6: postnatal day 6; VHP: vasa hyaloidea propria, TVL: tunica vasculosa lentis.

Supplementary table 1: detailed imaging conditions, organized by figure stating the objective magnification, additional optical zoom, pixel size and numerical aperture.

		objective magnification	additional optical magnification	X/Y pixel size (µm)	Z pixel size (µm)	Numerical aperture
Figure 1	all images	2X	2X	1.51	2	0.120
Figure2	E12.5 and E14.5		3.2X	0.944	1	
	P0 to P18		2.5X	1.21	1	
	P60		2X	1.51	2	
Figure3	all images		2.5X	1.21	1	
Figure 4	all images		2.5X	1.21	1	
Figure 5	all images		2X	1.51	2	
suppFig1	all images		2X	1.51	2	
suppFig2	a, b		6.3X	0.516	1	
	c to f		2X	1.51	2	
suppFig3	all images		2X	1.51	2	
suppFig4	all images		2.5X	1.21	1	
