# Supplementary material for: Three-dimensional characterization of developing and adult ocular vasculature in mice using in toto clearing
Source: Commun Biol. 2022 Oct 27;5:1135. doi: 10.1038/s42003-022-04104-2 (PMC9613908; doi:10.1038/s42003-022-04104-2)
Supplement: Supplementary file 3 — Description of Additional Supplementary Files [file 42003_2022_4104_MOESM3_ESM.pdf]

Description of Additional Supplementary Files

File name: Supplementary Video 1
Description: Video illustrates the animated 3D vascular and perivascular networks of developing and adult mice as shown in the figures. Annotations for each sample presented are incorporated directly in the video.
